# Serum oxidative stress influences neurological recovery after surgery to treat acutely worsening symptoms of compression myelopathy: a cross-sectional human study

**DOI:** 10.1186/s12891-019-2966-5

**Published:** 2019-12-07

**Authors:** Hiroshi Takahashi, Yasuchika Aoki, Junya Saito, Arata Nakajima, Masato Sonobe, Yorikazu Akatsu, Shinji Taniguchi, Manabu Yamada, Keita Koyama, Yuki Akiyama, Yasuhiro Shiga, Kazuhide Inage, Sumihisa Orita, Yawara Eguchi, Satoshi Maki, Takeo Furuya, Tsutomu Akazawa, Masao Koda, Masashi Yamazaki, Seiji Ohtori, Koichi Nakagawa

**Affiliations:** 1 Department of Orthopaedic Surgery, Toho University Sakura Medical Center, Center 564-1, Shimoshizu, Sakura, Chiba 285-8741 Japan; 2 Department of Orthopaedic Surgery, Eastern Chiba Medical Center, 3-6-2, Okayamadai, Togane, Japan; 3 Department of Orthopaedic Surgery, Chiba University Graduate School of Medicine, 1-8-1, Inohana, Chuoku, Chiba, Japan; 4 Department of Orthopaedic Surgery, Shimoshizu National Hospital, 934-5, Shikawatashi, Yotsukaido, Japan; 5 Department of Orthopaedic Surgery, St. Marianna University School of Medicine, 2-16-1, Sugao, Miyamaeku, Kawasaki, Japan; 6 Department of Orthopaedic Surgery, Faculty of Medicine, University of Tsukuba, 1-1-1, Tennodai, Tsukuba, Japan

**Keywords:** Compression myelopathy, Serum oxidative stress, Reactive oxygen metabolites, Surgical outcome

## Abstract

**Background:**

Recent reports indicate that oxidative stress induced by reactive oxygen species is associated with the pathobiology of neurodegenerative disorders that involve neuronal cell apoptosis. Here we conducted a cross-sectional study to evaluate serum levels of oxidative stress in cervical compression myelopathy.

**Methods:**

Thirty-six serum samples were collected preoperatively from patients treated for acutely worsening compression myelopathy (AM) and chronic compression myelopathy (CM). Serum levels of oxidative stress markers were evaluated by measuring derivatives of reactive oxygen metabolites (ROM), which reflect concentrations of hydroperoxides. ROM in healthy individuals range from 250 to 300 (U. CARR), whereas ROM >340–400 and > 400 define moderate and severe levels of oxidative stress, respectively. Difference of ROM by the cause of disorders whether cervical spondylotic myelopathy (CSM) or cervical ossification of longitudinal ligament (OPLL), correlations between ROM and patient age, body mass index (BMI), history of smoking, existence of diabetes were examined. Neurological evaluations according to Japanese Orthopaedic Association (JOA) scores were performed and correlated with ROM.

**Results:**

ROM increased to 349.5 ± 54.8, representing a moderate oxidative stress, in CM samples. ROM increased to 409.2 ± 77.9 in AM samples, reflecting severe oxidative stress which were significantly higher than for CM samples (*p* < 0.05). There was no significant difference by the cause of disorders (CSM or OPLL). ROM were significantly increased in AM serum samples from female patients versus AM male and CM patients (*p* < 0.05). There were no correlations between ROM and age, BMI, history of smoking, and existence of diabetes. A negative correlation between ROM and recovery rate of JOA score (R^2^ = 0.454, *p* = 0.047) was observed in the AM group.

**Conclusions:**

Although moderate oxidative stress was present in patients with CM, levels of oxidative stress increased in severity in patients with AM. These results suggest that postsurgical neurological recovery is influenced by severe oxidative stress in AM.

## Background

Cervical compression myelopathy is one of the most common spinal cord disorders affecting the elderly. It is well known that the mechanism of compression myelopathy is chronic compression of the spinal cord by degenerated discs, osteophytes, thickened ligamentum flavum, and ossification of the posterior longitudinal ligament [1]. Although a slow and stepwise decline in function is usually observed in compression myelopathy, a rapid progression of motor paralysis and paresthesia with mild or no trauma is occasionally observed. Acutely worsening symptoms of compression myelopathy result in severe neurological deficits with poor functional recovery [2]. To date, early surgical treatment is recommended as the only valid therapy for compression myelopathy, and the surgical outcome is relatively favorable [3]. However, in some cases, sufficient improvement in neurological function is not achieved [4]. Currently, an accurate prediction of the recovery rate of neurological function before surgical treatment is not possible. Furthermore, a subjective evaluation using the JOA score for example, is the only procedure available to assess the severity of neurological dysfunction [5]. Therefore, several biomarkers that reflect the degree of damage to the spinal cord and the severity of neurological symptoms would be useful.

Results of a recent study show that phosphorylated neurofilament subunit H (pNF-H) and tau protein, which reflect axonal damage to the spinal cord, become elevated in the cerebrospinal fluid (CSF) of patients with acutely worsening compression myelopathy [6, 7]. It was suggested that pNF-H in CSF may act as a biomarker to predict neurological recovery after surgical treatment. However, because CSF sample collection is strictly limited to patients who elect to undergo a myelogram, CSF samples are unsuitable for evaluating changes over time given associated ethical limitations. Therefore, more easily obtained serum biomarker is preferred.

Recent reports indicate that oxidative stress markers in serum are elevated in various neurodegenerative disorders such as Alzheimer’s disease, amyotrophic lateral sclerosis, and Parkinson’s disease [8, 9]. For orthopaedic lesions, it was reported that the ROM in serum reflected the severity of rheumatoid arthritis (RA) [10]. However, to our knowledge, no report has analyzed oxidative stress markers based on ROMs in the serum of patients with compression myelopathy. Therefore, we conducted a pilot cross-sectional study to determine the levels of oxidative stress in patients with compression myelopathy and to investigate any relationship of oxidative stress levels with clinical outcome.

## Methods

### Patients and sample selection

This study was approved by the Human Ethics Committee at Toho University Sakura Medical Center. Informed consent was obtained from all the patients from which we acquired preoperative serum samples during hematological examinations at our hospital from April 2015 to December 2017. Surgical treatment was recommended for all the patients with cervical compression myelopathy in this study. The cause of disorder included cervical spondylotic myelopathy (CSM) and cervical ossification of longitudinal ligament (OPLL). Cervical myelopathy was diagnosed from neurological findings, radiographs, and magnetic resonance images by three orthopaedic spine surgeons. The study exclusion criteria included patients who opted for conservative (nonsurgical) treatment; patients with RA, which can elevate oxidative stress [10]; patients who were diagnosed with cervical spondylotic radiculopathy, cervical spondylotic amyotrophy, trauma, or infection; and patients with a double lesion (cervical compression myelopathy and lumbar canal stenosis). No patients were excluded based on the severity of their myelopathy. Samples from patients with compression myelopathy were divided into two groups: those with acutely worsening symptoms (AM) and those with chronic symptoms (CM). If the JOA score of patients with cervical myelopathy decreased by 2 points or more within a 1-month period, patients were defined as having acutely worsening symptoms of compression myelopathy according to the previous reports [6, 7, 11]. In the patients those who were followed at our outpatient clinic, we checked them every 1-month. In the patients those who came to our hospital from the other clinics, we carefully listened to the current medical history from each patient and divided into AM and CM groups. All the patients were followed up for at least 1 year after surgery.

### Measurement of serum oxidative stress markers

A recently developed method for measuring derivatives of reactive oxygen metabolites (ROM) in serum, referred to as the d-ROM test, was used to evaluate reactive oxygen species (ROS) production not counteracted by anti-oxidative activity and thus representative of oxidative stress in serum. The d-ROM test is an integrative Free Radical Analytical System (FRAS, Wismarl, Italy) and was employed according to the manufacturer’s specifications [12]. The d-ROM test does not measure ROS directly, but rather detects hydroperoxide metabolites that are main derivatives of ROM. Hydroperoxides are converted into radicals that oxidize N, N-diethyl-para-phenylenediamine and can be detected spectrophotometrically using an automatic analyzer. ROM are expressed in arbitrary units called Carratelli units (U. CARR). If the candidate receives oxidative stress, ROS were produced in serum and ROM in serum increases. Generally, the normal range for ROMs is 250–300 U. CARR; > 340–400 U. CARR defines moderate oxidative stress; and > 400 U. CARR represents severe oxidative stress [13, 14]. All the serum samples were corrected during the hematological examinations at the time of about 1 month before surgery. The ROM in all serum samples collected were evaluated. As the first step, the difference of ROM by the cause of disorder (CSM or OPLL) were investigated. As the next step, the correlations between ROM and patient’s age, ROM and body mass index (BMI), ROM and sex, ROM and history of smoking (divided into three groups; never smoking, past history of smoking, and now smoking), ROM and existence of diabetes were examined.

### Evaluation of neurological improvement

The neurological status of all patients was evaluated by two orthopedic spine surgeons using the JOA scores for cervical myelopathy which ranged from 0 to 17 [5]. JOA scores were determined preoperatively at the time of serum sampling during a hematological examination and 1 year after surgery. The relationship between ROM in serum and JOA scores were evaluated. The recovery rate of JOA scores was calculated based on Hirabayashi’s method [15].

### Statistical analyses

Results are expressed as the mean ± standard deviation (SD). A Student’s t-test was used to evaluate the difference in ROM in serum between patients with AM and CM. A one-factor ANOVA with a post-hoc Tukey-Kramer test was used to evaluate the difference in ROM and cause of disorders (CSM or OPLL), sex, history of smoking, and existence of diabetes. A Mann–Whitney *U* test and Spearman’s correlation coefficient between ranked variables were used to evaluate preoperative JOA scores and any neurological recovery reflected by JOA scores. *P* < 0.05 was considered statistically significant. All statistical analyses were performed using SPSS (version. 21) software (IBM Corporation, Armonk, NY, USA).

## Results

### Patients and characteristics

A total of 52 patients with cervical myelopathy were screened for inclusion in this study. Serum samples were collected from 36 patients after 16 patients were deemed ineligible to participate because of either RA (3), trauma (4), infection (1), radiculopathy or amyotrophy (3), a double lesion (3), death (1), or because they dropped out of the study (1).

Patient characteristics are shown in Table 1. There was no significant difference in the mean age of the patients between the AM and CM groups. There was no statistical bias in the distribution of sex, smoking, and past history of diabetes between the AM and CM groups. The mean JOA score in the group of patients with AM was slightly worse than that in the group of patients with CM (*p* > 0.05). The cause of the underlying neurological disorder (CSM or OPLL), and the choice of surgical procedure: an anterior decompression and fusion (ADF), laminoplasty (LMP), or posterior decompression and fusion (PDF), were not significantly different between patients in the AM and CM groups.

**Table 1:** Patient characteristics in each group

	AM	CM	*p*
Number of cases	20	16	
Age (years)	67.2 ± 12.5 (35–86)	67.8 ± 10.3 (49–84)	0.878
BMI	24.6 ± 5.0 (17.5–36.0)	24.5 ± 3.7 (19.2–33.2)	0.924
Sex (Male / Female)	12 / 8	13 / 3	
Smoking (Never / Past / Now)	8 / 9 / 3	5 / 8 / 3	
Cause of disorder (CSM/OPLL)	17 / 3	11 / 5	
Past history of diabetes (+/−)	5 / 15	8 / 8	
JOA before surgery	9.8 ± 2.8 (5–14.5)	11.0 ± 2.2 (6–14)	0.142
Surgical procedure			
ADF	11	10	
LMP	6	4	
PDF	3	2	

*AM* Acutely worsening compression myelopathy, *CM* Chronic compression myelopathy, *CSM* Cervical spondylotic myelopathy, *OPLL* Ossification of longitudinal ligament, *JOA* Japanese Orthopaedic Association, *ADF* Anterior decompression and fusion, *LMP* Laminoplasty, *PDF* Posterior decompression and fusion. Data are the mean ± standard deviation (range)

### ROM in serum

**Fig. 1:**
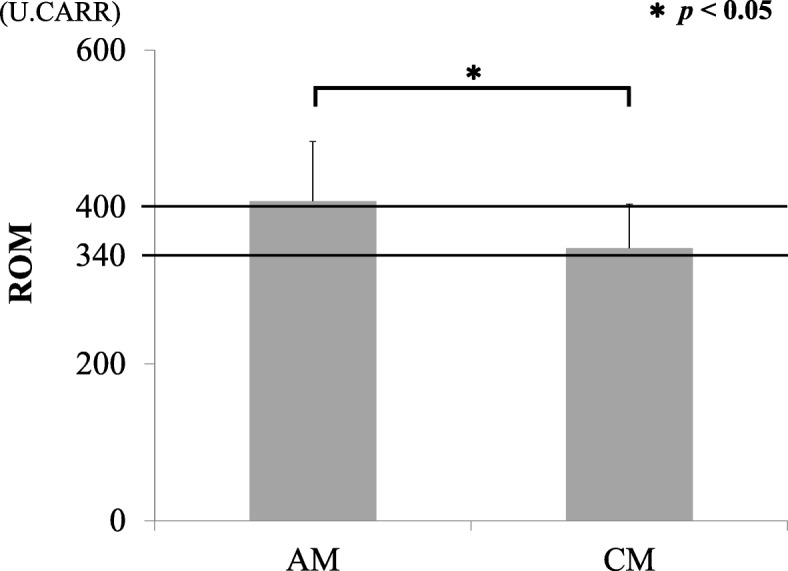
ROM in serum samples. AM: acutely worsening compression myelopathy, CM: chronic compression myelopathy. Threshold lines at 340 and 400 indicate moderate and severe oxidative stress, respectively. *P < 0.05 for comparisons using the Student’s t-test

**Fig. 2:**
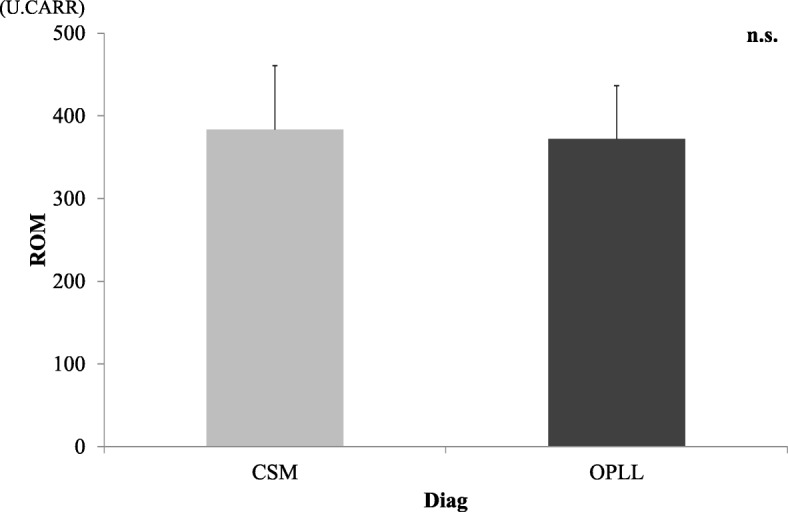
ROM and the cause of disorder (CSM or OPLL). There was no significant difference by CSM and OPLL

**Fig. 3:**
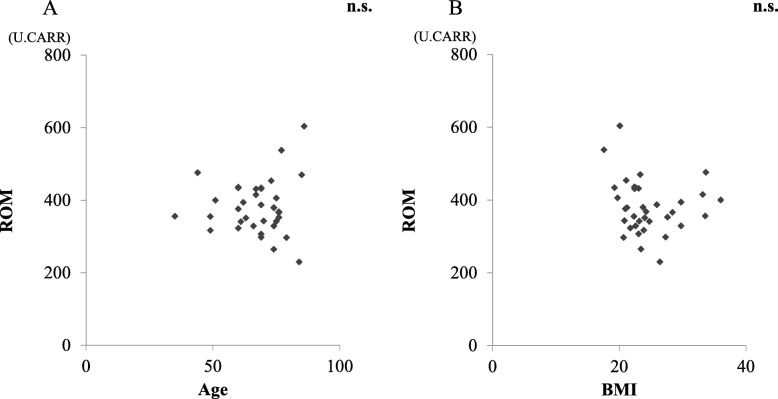
**a** ROM and age. **b** ROM and BMI. There was no correlation between ROM and age or BMI

**Fig. 4:**
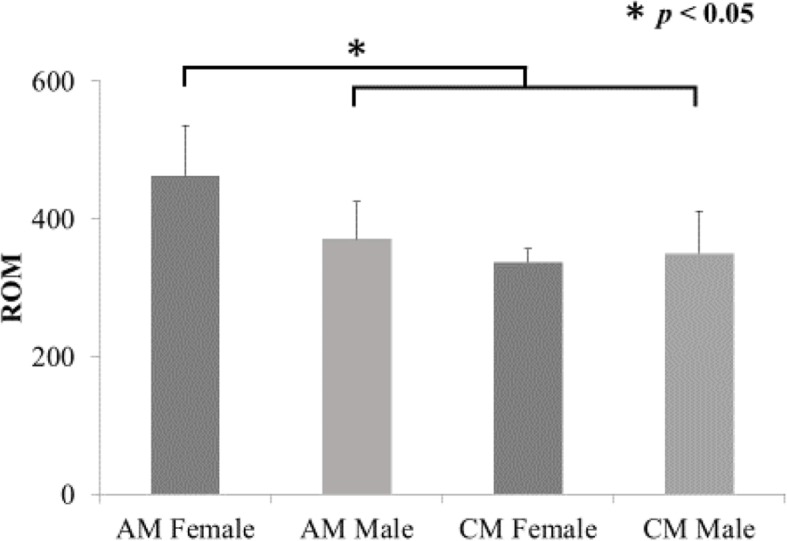
ROM and sex. A significant increase of ROM was observed in female of AM. *P < 0.05 for comparisons using a one-factor ANOVA

**Fig. 5:**
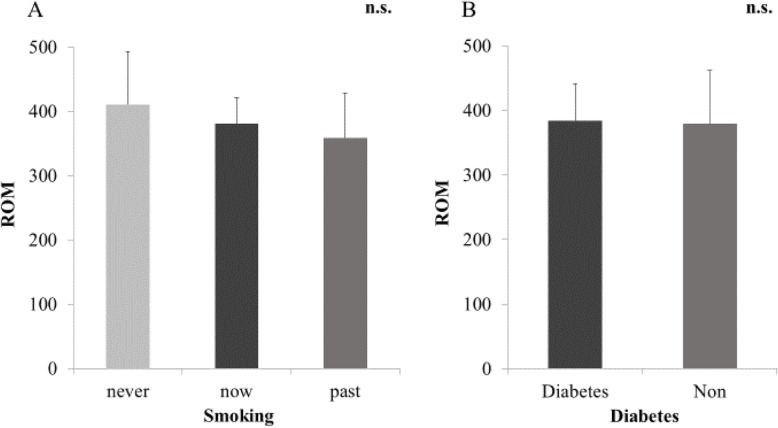
**a** ROM and history of smoking (never smoking, now smoking, and past history of smoking). **b** existence of diabetes. There was no difference between ROM and history of smoking or existence of diabetes

Mean ROM in the serum of patients in the AM and CM groups before surgery are shown in Fig. 1. ROM in the CM group were 347.6 ± 56.0, which was indicative of moderate oxidative stress in the serum. ROM in the AM group were 407.8 ± 76.1 and indicated a severe oxidative stress level that was significantly higher than that in the CM group (*p* < 0.05). There was no significant difference between ROM and the cause of neurological disorders (CSM/OPLL) (Fig. 2). There were no significant correlations between ROM and age or BMI (Fig. 3). There was a significant increase in ROM in the serum of female patients especially in the AM group (*p* < 0.05, Fig. 4). There was also no significant difference between ROM and past history of smoking or presence of diabetes (Fig. 5).

### Neurological recovery and the relationship with ROM

**Fig. 6:**
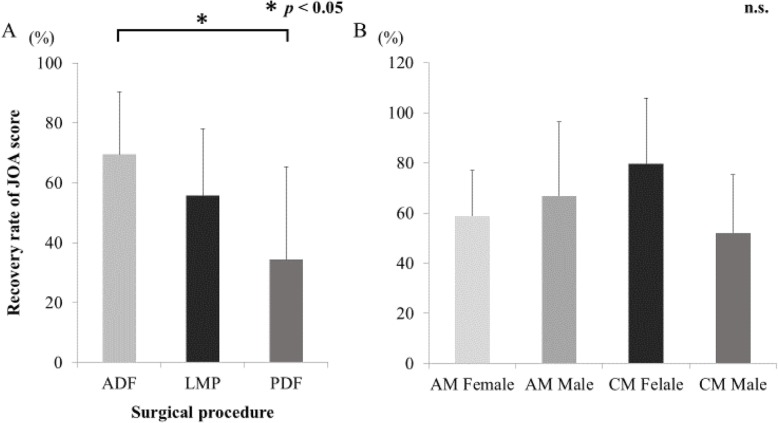
**a** ROM and surgical procedure. ADF: anterior decompression and fusion, LMP:laminoplasty, PDF: posterior decompression and fusion. A significant difference among the three surgical procedure was observed (one-factor ANOVA, *p* < 0.05). **b** ROM, AM/CM, and sex. AM female: 8, AM male: 12, CM female: 3, CM male: 13 patients. There was no significant difference among those four groups

**Fig. 7:**
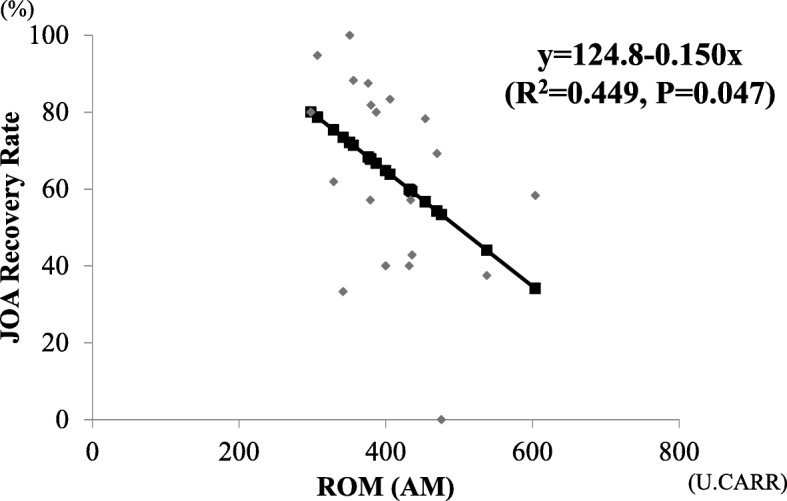
Negative correlation between ROM and recovery rates based on the JOA score in the AM group

Changes in JOA scores after surgery are shown in Table 2. Favorable neurological improvement or recovery was achieved in both groups. The recovery rate of JOA score was slightly higher in patients in the AM group than in the CM group, although the difference between them was not significant. The recovery rate of JOA score depending on the surgical procedure are shown to Fig. 6. There was significant difference among the three surgical procedure of ADF (21 cases), LMP (10 cases), and PDF (5 cases) (one factor ANOVA, *p* < 0.05). There was no significant difference between sex and recovery rate of JOA score. In the analysis of AM group, a negative correlation was observed between ROM in serum and recovery rate of JOA scores (*R*^2^ = 0.449, *p* = 0.047; Fig. 7). On the other hands, in the analysis of CM group, there was no correlation between ROM in serum and recovery rate of JOA scores (data not shown).

**Table 2:** The recovery of JOA score

	AM	CM	*p*
JOA score before surgery	9.8 ± 2.8 (5–14.5)	11.0 ± 2.2 (6–14)	0.142
JOA score 1 year after surgery	14.5 ± 1.8 (9.5–17)	14.2 ± 2.1 (10–17)	0.629
Recovery rate of JOA score	63.5 ± 25.4 (0–100)	57.2 ± 25.5 (20–100)	0.461

*AM* Acutely worsening compression myelopathy, *CM* Chronic compression myelopathy, *JOA* Japanese Orthopaedic Association. Data are the mean ± standard deviation (range)

### A representative case

**Fig. 8:**
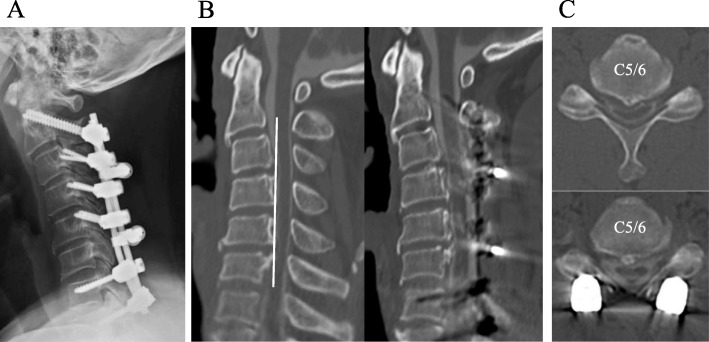
A representative case. **a** A lateral radiograph after surgery shows posterior decompression and fusion surgery were performed. **b** A reconstructed sagittal CT myelograph before and 1 year after surgery. The white line represents the K-line. The OPLL in this case is K-line (−). **c** An axial CT myelograph at the C5/6 level. Sufficient decompression was obtained

A representative case of a 44-year-old male who was diagnosed with cervical OPLL was assessed for our study (Fig. 8). The patient was obese with a body mass index of 33.7 kg/m^2^ and had been diagnosed with diabetes in the past. Prior to surgery, his ROM in serum were 476 U. CARR, which was indicative of a severe level of oxidative stress. Three months after PDF surgery, his JOA score improved from 9.5 to 12.0. Nevertheless, his neurological symptoms worsened despite a postoperative CT myelograph that confirmed his spinal cord decompression was successful. The patient’s JOA score 1 year after surgery decreased to 9.5 and his neurological recovery rate based on the JOA score was 0%.

## Discussion

To our knowledge, the present study is the first to determine the levels of oxidative stress markers in the serum of patients with compression myelopathy. Generally, biological markers of oxidative stress are molecules that are modified via interactions with ROS. As previously stated, the direct measurement of ROS in serum is difficult because of their biochemical instability [14]. Thus, an assay to detect a more stable class of ROM, or hydroperoxides, has been developed. In fact, measuring ROM, a proxy for ROS production, has already been recognized as useful for the evaluation of oxidative stress levels in a multitude of diseases including, but not limited to, obesity, metabolic syndrome, asthma, and RA [10, 16, 17]. In addition, although the pathogenesis of compression to the spinal cord is a little different between CSM and OPLL, there were no significant difference by the cause of disorders (CSM or OPLL) in this study that suggest that OPLL does not concern to the elevation of ROM.

According to past reports, markers of oxidative stress increase with increasing age, female gender, obesity, and diabetes [12, 14, 16] and endogenous oxidative stress has been implicated. The markers of oxidative stress in serum were increased in females in the AM group compared with males in the AM, males in the CM, and females in the CM group, a finding also in partly supported by a previous study [12]. However, there were no significant difference between the sex and the recovery rate of JOA score. To date, we have no idea to speculate this phenomenon. Further investigation will be needed to explain this phenomenon. On the other hands, in our study, there were no correlation between ROM and age, BMI, history of smoking, and existence of diabetes that may indicates those factors less concern to the elevation of oxidative stress.

In contrast, the level of oxidative stress based on serum analyses increases when there is an acute spinal cord injury, Alzheimer’s disease, amyotrophic lateral sclerosis, and Parkinson’s disease [9, 18, 19]. Reports of oxidative stress in the presence of neurodegenerative diseases suggests that there exists the neurogenic oxidative stress in which neuronal cell apoptosis may be involved. In this study, although ROM in the serum of patients in the CM group showed evidence of a moderate level of oxidative stress, patients in the AM group had ROM indicative of a significantly more severe level of oxidative stress. This may, at least in part, reflect more oxidative stress resulting from worsening neurodegeneration in the AM group. Furthermore, in the present study, high ROM in the AM group are reflective of a poor surgical outcome. Neuronal apoptosis may be in partly more serious in patients in the AM group with high ROM considering the favorable neurological recovery of CM. In addition, the recent report has indicated that the ischemia-reperfusion phenomenon concerns the surgical outcome in AM as the major pathogenesis [20]. Our results may reflect that the ischemia-reperfusion injury is also more serious in patients in the AM group with high ROM. Ultimately, the results of the present study suggest that the surgical outcome may be poor in patients with high ROM, like those in the AM group or the representative case. Thus, the results help us to explain during the time of informed consent, before surgery, that the surgical outcome may be inferior in such patients. Nevertheless, the ratio of endogenous oxidative stress to neurogenic oxidative stress is unclear. In addition, it is also unclear as to whether endogenous or neurogenic factors are strongly influential in compression myelopathy. Further basic and clinical investigations will be needed.

The present study has some limitations. First, the sample size was small, and the severity of myelopathy and the selection of the surgical procedure could contain some bias. Although our previous report has indicated that the recovery rate based on the JOA score was significantly higher in the AM group than in the CM group, unexpectedly, there were no significant differences in our study [6]. In that study, the main surgical treatments were posterior procedures such as LMP and PDF. However, we mostly chose ADF for the patients in our study. For K-line (−) OPLL, ADF or PDF was considered suitable surgical treatment based on JOA scores to assess recovery compared with LMP [15]. In addition, in cases of CSM, ADF is recommended for the treatment of multilevel cervical myelopathy compared with LMP [21]. In fact, the result of this study indicated that the recovery rate of JOA score is significantly higher in ADF. The choice of anterior surgery in our study may have resulted in favorable surgical outcomes in patients in the CM group. Further investigations with single surgical procedures will be needed to clarify the comparison of surgical outcome between AM and CM groups. Second, the definition of AM and CM is subjective because the distinction is only due to the neurological findings. CM contain both the sub-acute phase and real chronic phase. That is the other reason that there was no significant difference in the recovery rate of JOA score between AM and CM. It was unable to speculate surgical outcome of AM/CM only by ROM before surgery because oxidative stress also increased in CM. The definition of CM also may explain this unexpected phenomenon. We speculate that ROM increases until the sub-acute phase and gradually decreases to the normal level if the patient become the real chronic phase. Nevertheless, to our knowledge, there are no biomarkers that distinguish acute, sub-acute, and chronic phase of myelopathy accurately. Further investigation about ROM in combination with the other useful biomarkers will be expected. Third, we lacked data on ROM in serum over time. In this pilot study, we corrected the serum samples only at the time before surgery to clarify whether the ROM before surgery can speculate the surgical outcome. However, the collection of serum samples after surgery is easier than sampling CSF [6]. In the near future, we plan to conduct the observational study to investigate longitudinal changes in ROM after surgery. Such an approach could be used to determine the ratio of neurogenic oxidative stress associated with compression myelopathy. We speculate that if ROM improve postoperatively, and there is a correlation between them and the recovery rate assessed using JOA scores, oxidative stress levels with a neurogenic origin may be improved by surgery in patients with compression myelopathy. If our hypothesis is proven, it could result in the more effective use of antioxidant medications. Fourth, we don’t investigate the patient-based outcomes like SF-36, EQ-5D, and JOA CMEQ in this study. Although the patient-based outcome is important, the major purpose of this study is to evaluate the recovery of myelopathy. However, those patient-based outcomes may include the residual neck pain or psychogenic factor after surgery. To simplify the result, we investigated only the JOA score. Fifth, details of the mechanism underpinning the elevation of oxidative stress levels in patients with compression myelopathy remain unclear. In addition to neuronal apoptosis [8] and neurodegenerative changes that can result in increased oxidative stress levels, a recent report has indicated that modic type endplate changes in vertebrae of the lumbar spine and disc degeneration could also cause inflammation and play a pathologic role in compression myelopathy [22].

## Conclusions

Although moderate levels of oxidative stress were present in patients with chronic myelopathy, serum markers of oxidative stress levels were classified as severe in patients with acutely worsening myelopathy. Severe levels of oxidative stress in patients with acutely worsening myelopathy can influence neurological recovery after surgery. However, further investigation will be needed to collect longitudinal measures of ROM over time.

## Data Availability

The datasets used during the current study are available from the corresponding author on reasonable request.

## Abbreviations

ADF: Anterior decompression and fusion
AM: Acute worsening compression myelopathy
BMI: Body mass index
CM: Chronic compression myelopathy
CSF: Cerebrospinal fluid
CSM: Cervical spondylotic myelopathy
JOA: Japanese Orthopaedic Association
LMP: Laminoplasty
OPLL: Ossification of longitudinal ligament
PDF: Posterior decompression and fusion
pNF-H: Phosphorylated neurofilament subunit H
RA: Rheumatoid arthritis
ROM: Reactive oxygen metabolites
ROS: Reactive oxygen species
